# Green Synthesis and Investigation of Antimicrobial Activity and Compressive Resilience of Glass Ionomer Cement Modified With Zirconia Nanoparticles: An In Vitro Study

**DOI:** 10.7759/cureus.62837

**Published:** 2024-06-21

**Authors:** Khushi Jain, Jessy Paulraj, Subhabrata Maiti, Rajeshkumar Shanmugam

**Affiliations:** 1 Department of Pedodontics and Preventive Dentistry, Saveetha Dental College and Hospitals, Saveetha Institute of Medical and Technical Sciences, Saveetha University, Chennai, IND; 2 Department of Prosthodontics, Saveetha Dental College and Hospitals, Saveetha Institute of Medical and Technical Sciences, Saveetha University, Chennai, IND; 3 Nanobiomedicine Lab, Centre for Global Health Research, Saveetha Medical College and Hospitals, Saveetha Institute of Medical and Technical Sciences, Saveetha University, Chennai, IND

**Keywords:** restorative dentistry, compressive resilience, antimicrobial, zirconia nanoparticles, modified gic, green synthesis

## Abstract

Background

Glass ionomer cement (GIC) serves as a crucial biomaterial in dental restoration, offering applications in filling, lining, and adhesive procedures. Nevertheless, its mechanical properties often fall short, particularly in regions subjected to considerable stress. To address this issue, zirconia nanoparticles are incorporated at specific levels.

Aim

To assess the antimicrobial efficacy and compressive resilience of GIC modified with zirconia nanoparticles synthesized through green synthesis methods.

Material and methods

Zirconia nanoparticles were synthesized via a green method utilizing aloe vera extract in solvent form. These nanoparticles were then mixed into GIC at different concentration levels. Group I incorporated zirconia nanoparticles at a concentration of 3%, Group II at 5%, and Group III at 10%, while Group IV was the control, consisting of traditional GIC. Following that, samples were prepared and underwent characterization through various analytical techniques. The ability to inhibit microbial growth and the compressive resilience of the groups were examined. Microbial inhibition against the bacterial strains was assessed through minimum inhibitory concentration (MIC), and the ability to withstand compression was gauged by measuring the maximum force the specimen could endure before fracturing. Data underwent analysis with Statistical Package for the Social Sciences (IBM SPSS Statistics for Windows, IBM Corp., Version 24.0, Armonk, NY). Repeated measures of analysis of variance (ANOVA) were utilized to gauge average MIC values and compressive strength. Following this, Tukey's post hoc test was employed for pairwise comparisons.

Results

The findings indicated, incorporating zirconia nanoparticles into GIC led to an improvement in its antimicrobial effectiveness, with a noticeable enhancement observed as the weight percent (% wt) of the additive increased. This improvement was notably noticeable in its effectiveness against *Streptococcus mutans* and *Lactobacillus*, exceeding that of the control with a noteworthy distinction. Furthermore, there were significant enhancements in compressive strength, in Group I (180.48 ± 1.02), Group II (191.25 ± 0.52), and Group III (197.52 ± 0.75), compared to Group IV (167.22 ± 1.235), with significant disparities (p < 0.05).

Conclusion

The research illustrates that introducing green-synthesized zirconia nanoparticles into GIC leads to heightened bactericidal potency and compressive resilience when contrasted with the control group (Group IV). Notably, the highest concentration of 10% demonstrated the most favourable antimicrobial attributes alongside enhanced strength. Consequently, integrating green-synthesized zirconia nanoparticles into GIC holds potential as a proficient material. In future studies, there should be an exploration of molecular chemistry and bonding mechanisms to enhance our comprehension of its capabilities.

## Introduction

Glass ionomer cement (GIC) serves as a dental restorative material in dentistry, utilized for filling and luting purposes. It comprises silicate glass powder and polyacrylic acid, forming an ionomer [1]. Its primary function is to prevent dental caries, owing to its strong adhesive bond properties with tooth structure, facilitating a tight seal between the tooth's internal structures and its surroundings. Recent years have witnessed several innovative enhancements to GIC properties while streamlining its application. Unlike earlier versions, these newer systems are more user-friendly for various age groups. Moreover, these enhanced GICs purport to tackle long-standing issues like poor fracture resistance, which have hindered their clinical utility [2]. Similar to other dental materials GICs also have weaknesses, primarily sensitivity to moisture and low initial strength. Efforts have been made to refine the properties of initial GIC and alleviate the mentioned weaknesses. These endeavours involve alterations in the structure of the glass ionomer powder and polyacrylic acid, resulting from evident disparities in chemical composition, physical properties, and the utilization of different commercial materials [3]. One approach to bolstering the mechanical characteristics of GIC is by adding metal fillers.

The green synthesis of metal oxide nanomaterials has emerged as a prominent research area in nanobiotechnology, offering advantages such as reduced reliance on high temperatures, energy, pressure, and toxic chemicals. Biosynthesis of nanoparticles presents a cost-effective and environmentally friendly alternative to traditional chemical and physical methods. Plant-mediated synthesis, a green chemistry approach, bridges nanotechnology with plants [4]. Utilizing biomaterials in nanoparticle synthesis represents a cutting-edge focus in modern nanotechnologies. Natural herbal products suit the new era of research in recent years for producing metal oxide nanoparticles aiming to mitigate potential risks associated with toxic chemicals, ensuring a safe and environmentally friendly approach [5].

Zirconium nanoparticles have garnered attention in various syntheses, boasting exceptional fracture toughness, high tensile strength, and hardness [6]. The adoption of plant materials in the production of zirconia nanoparticles has garnered attention for its eco-friendly, uncomplicated, swift, non-toxic, and cost-effective approach, presenting a straightforward method for environmentally conscious nanoparticle synthesis [7,8]. Sundrarajan et al. showcased the antimicrobial and antifungal properties of zirconia nanoparticles mediated by aloe vera aqueous extract [9]. Therefore, the research aims to compare and evaluate the antibacterial properties and compressive durability of GIC enhanced with zirconia nanoparticles synthesized from green-origin methods against traditional GIC formulations. The null hypothesis proposes that these modifications will not display antibacterial properties or affect compressive strength when contrasted with traditional GIC.

## Materials and methods

Determination of sample size and materials utilized

The research was conducted at Saveetha Research Centre, Saveetha University, India. Ethical clearance was obtained for this in-vitro investigation from the Institutional Review Board, under the identifier SRB/SDC/UG-2047/23/PEDO/070. The sample size was calculated using the G*Power software (Heinrich-Heine-Universität Düsseldorf, Düsseldorf, Germany), targeting power of 0.95 with an effect size of 0.6 necessitating 48 samples in total. The GIC utilized in the study was from GC Corporation, Japan. Aloe vera dried leaves were procured from Annai Aravindh Herbals Pvt Ltd., Chennai, India. Zirconium oxide was obtained from Sigma-Aldrich Chemicals Private Limited., Bengaluru, India.

Preparation of green-mediated zirconia nanoparticle

**Figure 1:**
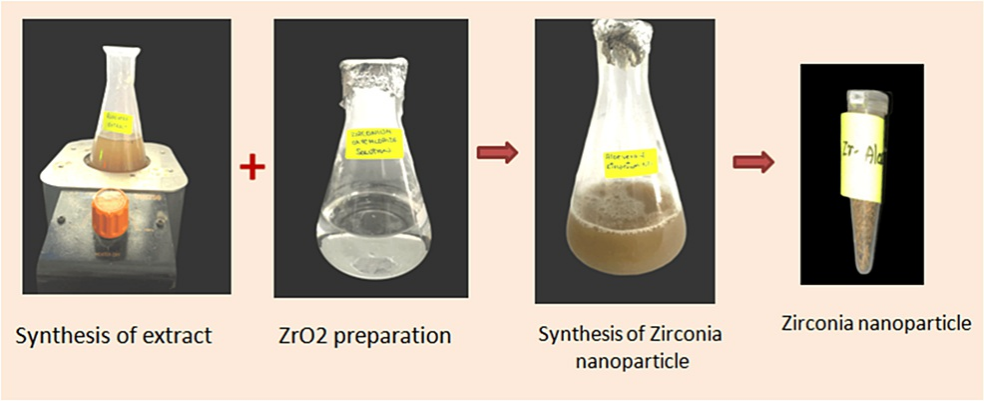
Green synthesis of zirconia nanoparticles ZrO_2_: zirconium dioxide

A gram of dried aloe vera powder was mixed with 100 mL of distilled water and heated to 40-50 degrees Celsius for 5-10 minutes. After cooling, the solution was filtered using Whatman No. 1 filter paper (Cytiva, Marlborough, USA). Zirconium oxychloride octahydrate, at a concentration of 20 millimolars, was dissolved in 50 mL of distilled water. Aloe vera extract (50 mL) was added to 50 mL of the zirconium precursor solution, and the mixture was stirred continuously at 340-360 RPM. The mixture was allowed to stand overnight until a colour change occurred (Figure 1).

Finally, the solution was lyophilized in a freeze dryer for 48 hours at -92°C to obtain a fine powder, aiming to enhance nanoparticle stability while preserving biochemical properties.

Incorporation technique of green-mediated zirconia nanoparticle into the GIC

The GIC was infused with zirconia nanoparticles at concentrations of 3% (Group I), 5% (Group II), and 10% (Group III), with conventional GIC serving as the control in Group IV. The combination of conventional GIC powder and zirconia nanoparticles was thoroughly blended using a vortex machine, following established mixing protocols [10]. Following this, the powdered mixture was mixed with the liquid component of the GIC according to the recommended powder-to-liquid ratio provided by the manufacturer.

Investigating with Fourier transform infrared (FTIR) analysis

This analysis was employed to evaluate the impact of zirconia nanoparticle incorporation. Measurements were performed using a Nicolet iS10 instrument from Thermo Fisher Scientific, Waltham, USA operating in the spectral range of 600-3500 cm^-1^ with a resolution of 4 cm^-1^, and employing 32 scans per spectrum to analyze chemical bonds.

Scanning electron microscopy (SEM) analysis

The structural characteristics of all samples were investigated using SEM (Jeol-JSM IT-800, Germany). Before analysis, each specimen underwent sputter coating. The coating procedure lasted for 180 seconds.

Elemental analysis by energy dispersive X-ray (EDX)

Cement samples were formulated in the form of powder and subjected to examination with the EDX (EDX-7200; Shimadzu, Kyoto, Japan). The findings were expressed as percentages of element weight, with precision up to two decimal places.

Evaluation of antibacterial activity

**Figure 2:**
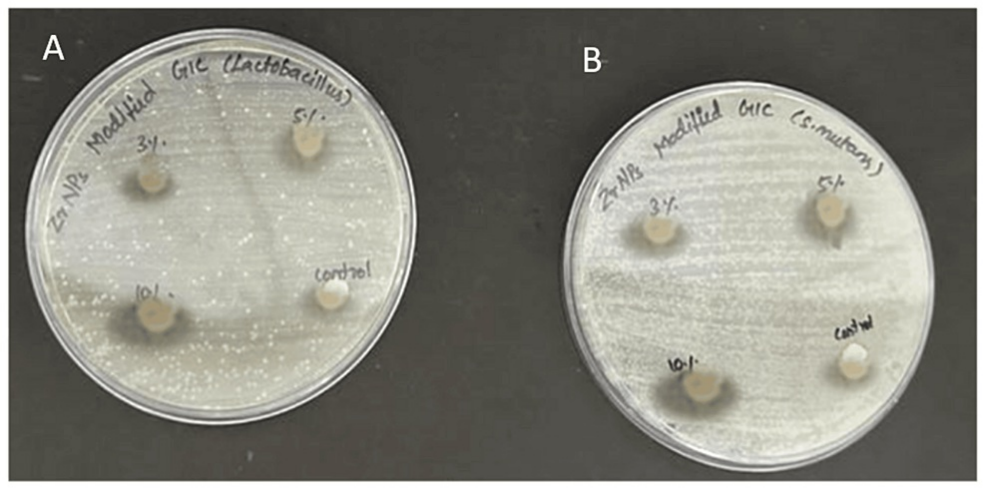
Antibacterial activity of zirconia nanoparticle-modified GIC (A) *Lactobacillus*; (B) *Streptococcus mutans* GIC: glass ionomer cement

Green-mediated zirconia nanoparticles were added into GIC at 3%, 5%, and 10%, following the manufacturer's instructions for blending with the polyacrylic acid-based liquid. The ingredients were blended with a plastic spatula until a uniform paste was obtained. The resulting cement, by the specified weight percentages, was placed into stainless steel moulds (6 mm x 2 mm) covered with a celluloid strip, and pressed under manual compression against a glass slab. After a 30-minute setting period, the sample was left undisturbed for 24 hours. The lab-grown cultures of *Streptococcus mutans* and *Lactobacillus acidophilus *were incubated on Mueller-Hinton agar for 24 hours at 37°C, and then moved to broth. Each group had 12 specimens, divided equally between *S. mutans* and *L. acidophilus*, with a total of 48 samples. Mueller-Hinton agar broth was evenly distributed into all wells, followed by the addition of bacterial suspensions (Figure 2).

Minimum inhibitory concentration (MIC) assays were conducted for groups modified at 3%, 5%, and 10%, along with unmodified GIC as the control. Each assay was repeated 12 times per group for reliability. Samples were observed for one to five hours, and cell death percentages were determined using an enzyme-linked immunosorbent assay (ELISA) reader at 540 nm.

Compressive strength evaluation

Compressive strength evaluation followed ISO 9917-1:2007 standards. Each group included 12 specimens, amounting to 48 in total, created using cylindrical moulds (4mm x 6mm). Cocoa butter was applied to the moulds to facilitate sample retrieval. The material was filled into the moulds, covered, and gently pressed to eliminate air bubbles. After 30 minutes, the specimens were retrieved and kept in distilled water for a day. Each sample was positioned vertically in an Instron universal testing machine (ElectroPuls, India) and compression was exerted at a speed of 0.5 millimetres per minute until fracture occurred, and the data was recorded.

Statistical analysis

Analysis of variance (ANOVA) followed by Tukey’s test was utilized to analyze mean differences, with Statistical Package for the Social Sciences (IBM SPSS Statistics for Windows, IBM Corp., Version 24.0, Armonk, NY) employed for statistical analysis. A significance threshold of p < 0.05 was established to ascertain statistical significance.

## Results

Characterization of green-mediated zirconia nanoparticle-modified GIC

**Figure 3:**
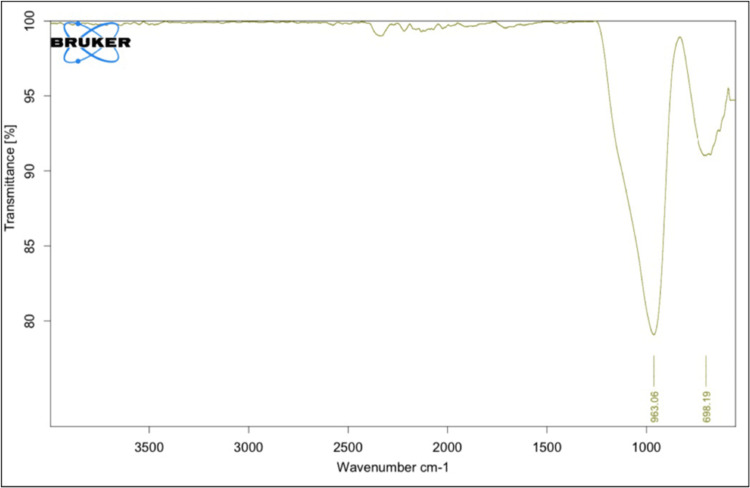
FTIR pattern of zirconia-modified GIC FTIR: Fourier transform infrared; GIC: glass ionomer cement

The FTIR spectrum of the zirconia-modified samples was noticed within the spectrum of 600-3500 cm^−1^. A notable absorption, characterized by a peak at 698.19 cm^−1^, was identified, credited to the zirconium oxide oscillation of the tetragonal structure. The width of this band indicated the nanocrystalline nature of the zirconia powders. Additionally, an absorption peak at 963.06 cm^−1^ was observed, corresponding to O-H bonding (Figure 3).

**Figure 4:**
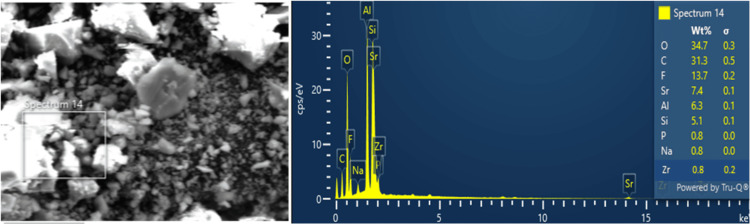
EDX spectrum of zirconia-modified GIC GIC: glass ionomer cement; EDX: energy dispersive X-ray

The EDX analysis detected the existence of several elements, constituting oxygen (O) at 34.7% of the composition, followed by carbon (C) at 31.3%. Fluorine (F) was detected at 13.7%, while aluminium (Al) accounted for 6.3% and silica for 5.1% of the composition. Minor elements such as phosphorus, sodium, and zirconia at 0.8% were also identified through EDX analysis (Figure 4).

**Figure 5:**
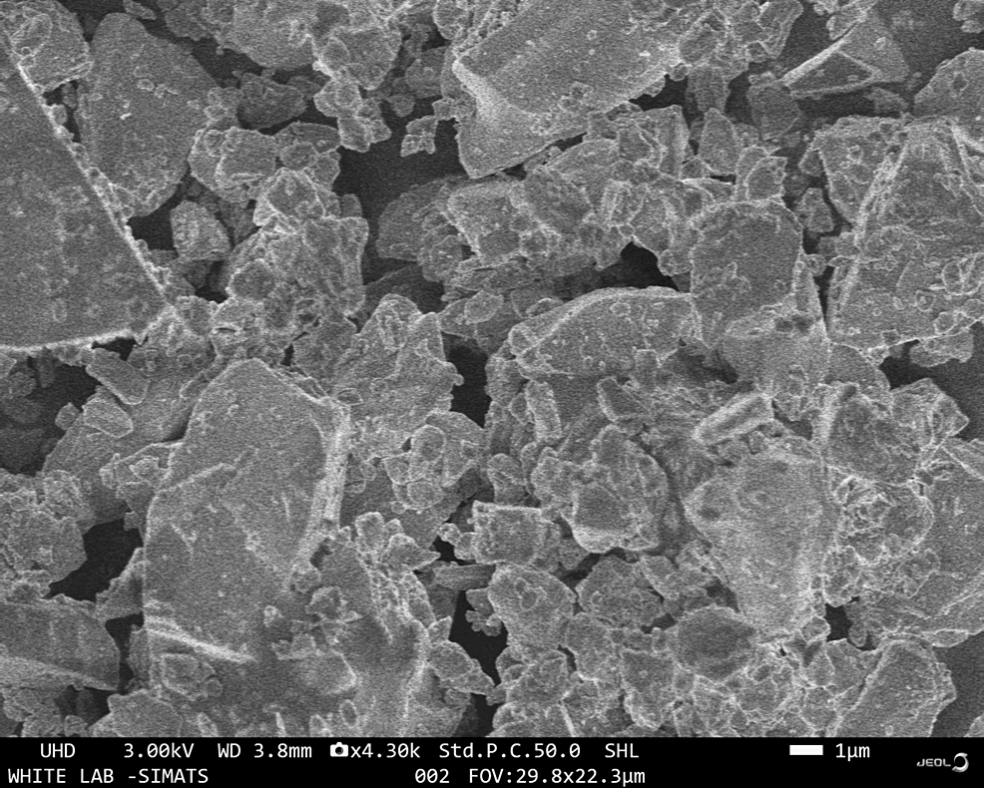
SEM micrograph under magnification of 30,000X for zirconia-modified GIC specimen SEM: scanning electron microscopy; GIC: GIC: glass ionomer cement

The SEM images revealed a non-uniform glass framework with an irregular scattering of particles, alongside a more uniform microstructure. This observation underscores the possible improvement in mechanical characteristics and overall functionality resulting from the material's nano-modification (Figure 5).

Evaluation of antimicrobial activity against *S. mutans*

**Figure 6:**
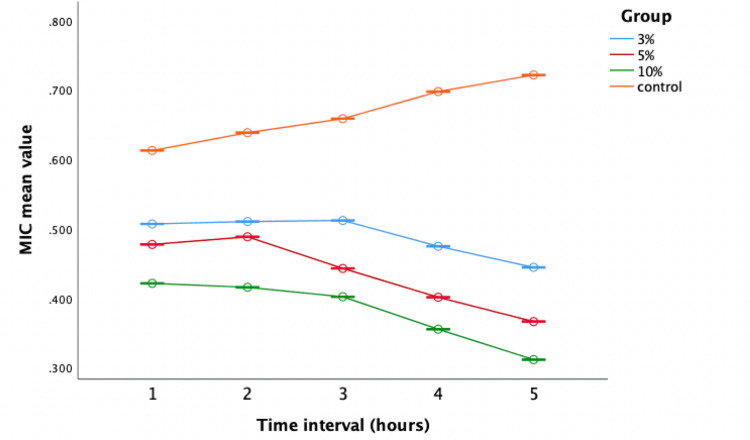
Antimicrobial efficacy against Streptococcus mutans The X-axis represents the time interval (one to five hours) and the Y-axis represents the mean values (0.300 to 0.800). MIC: minimum inhibitory concentration

The modified groups outperformed the control (Group IV) significantly. In particular, the 10% concentration (Group III) exhibited potent antimicrobial properties, suggesting increased efficacy with elevated weight percentages (Figure 6).

The one-way ANOVA indicated notable differences in antibacterial effectiveness across various time intervals, consistently demonstrating that Group III exhibited the minimum average values (0.422, 0.376, 0.321, 0.284, 0.238), signifying superior efficiency. Conversely, Group IV displayed greater mean values (0.563, 0.565, 0.597, 0.634, 0.676) throughout, suggesting increased bacterial growth and reduced antibacterial activity (Table 1).

**Table 1:** Comparison of the mean value of Streptococcus mutans based on different time intervals * Significant at 0.05, p-value derived from one-way analysis of variance (ANOVA).

Time Intervals (hours)	Groups	N	Mean± Std. Deviation	Std. Error	95% Confidence Interval for Mean		F value	P value
					Lower Bound	Upper Bound		
First	G1 (3%)	6	0.467±0.00083	0.00034	0.466	0.468	37583.52	0.001*
	GII (5%)	6	0.463±0.00083	0.00034	0.462	0.464		
	GIII (10%)	6	0.422±0.00083	0.00034	0.421	0.423		
	GIV (control)	6	0.563±0.00040	0.00016	0.562	0.563		
Second	G1 (3%)	6	0.444±0.00051	0.00021	0.443	0.444	60212.47	0.001*
	GII (5%)	6	0.422±0.00098	0.00040	0.421	0.423		
	GIII (10%)	6	0.376±0.00083	0.00034	0.375	0.377		
	GIV (control)	6	0.565±0.00081	0.00033	0.564	0.566		
Third	G1 (3%)	6	0.402±0.00051	0.00021	0.402	0.403	86643.07	0.001*
	GII (5%)	6	0.366±0.00083	0.00034	0.365	0.367		
	GIII (10%)	6	0.321±0.00132	0.00054	0.320	0.323		
	GIV (control)	6	0.597±0.00116	0.00047	0.596	0.599		
Fourth	G1 (3%)	6	0.375±0.00051	0.00021	0.374	0.375	150157.43	0.001*
	GII (5%)	6	0.312±0.00098	0.00040	0.311	0.313		
	GIII (10%)	6	0.284±0.00081	0.00033	0.283	0.285		
	GIV (control)	6	0.634±0.00147	0.00060	0.632	0.635		
Fifth	G1 (3%)	6	0.321±0.00083	0.00034	0.320	0.322	347711.39	0.001*
	GII (5%)	6	0.265±0.00063	0.00025	0.264	0.265		
	GIII (10%)	6	0.238±0.00083	0.00034	0.237	0.239		
	GIV (control)	6	0.676±0.00103	0.00042	0.675	0.677		

Tukey's honestly significant difference (HSD) test confirmed significant differences between Group IV and the others, highlighting the enhanced antimicrobial activity of zirconia-modified groups (Table 2).

**Table 2:** Pairwise comparison of antimicrobial efficacy of Streptococcus mutans among all groups * Significant at the 0.05 level; the error term is mean square (error)=2.257E-7.

Pairwise Comparison	Mean Difference	Std. Error	P value	95% Confidence Interval	
				Lower Bound	Upper Bound
3% vs 5%	0.036	0.00027	0.001*	0.035	0.037
3% vs 10%	0.073	0.00027	0.001*	0.072	0.074
3% vs control	-0.205	0.00027	0.001*	-0.206	-0.204
5% vs 10%	0.037	0.00027	0.001*	0.036	0.037
5% vs control	-0.241	0.00027	0.001*	-0.242	-0.240
10% vs control	-0.278	0.00027	0.001*	-0.279	-0.277

Evaluation of antibacterial activity against *L. acidophilus*

**Figure 7:**
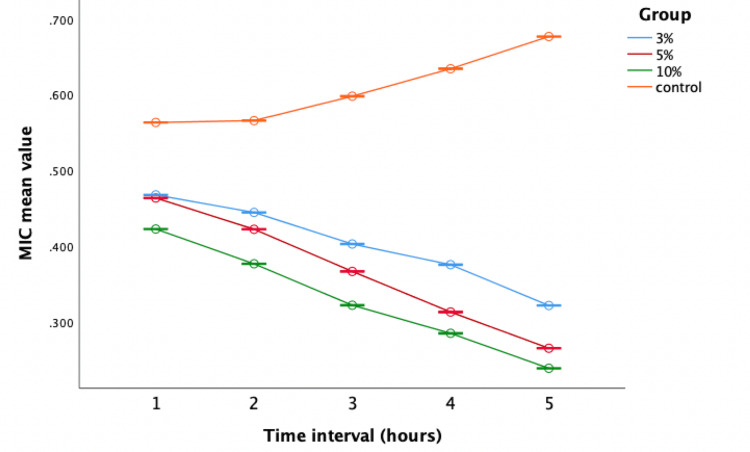
Antimicrobial efficacy against Lactobacillus The X-axis represents the time interval (one to five hours) and the Y-axis represents the mean values (0.300 to 0.700). MIC: minimum inhibitory concentration

The repeated measures of ANOVA analysis revealed enhanced bactericidal potency of zirconia nanoparticle-altered GIC groups (Groups I, II, and III) against *Lactobacillus *when compared to the control group (Figure 7).

One-way ANOVA revealed notable differences in bactericidal activity across the time intervals against *Lactobacillus.* Group III consistently exhibited the lowest mean values, indicating superior antibacterial potential (Table 3).

**Table 3:** Comparison of the mean value of Lactobacillus based on different time intervals * Significant at 0.05, p-value derived from one-way analysis of variance (ANOVA).

Time Intervals (Hours)	Groups	N	Mean± Std. Deviation	Std. Error	95% Confidence Interval for Mean		F value	P value
					Lower Bound	Upper Bound		
One	G1 (3%)	6	0.507±0.0006	0.0002	0.506	0.507	42161.81	0.001*
	GII (5%)	6	0.477±0.0012	0.0005	0.476	0.478		
	GIII (10%)	6	0.421±0.0008	0.0003	0.420	0.422		
	GIV (control)	6	0.612±0.0010	0.0004	0.611	0.613		
Two	G1 (3%)	6	0.510±0.0008	0.0003	0.509	0.511	40936.63	0.001*
	GII (5%)	6	0.488±0.0005	0.0002	0.487	0.489		
	GIII (10%)	6	0.415±0.0016	0.0006	0.414	0.417		
	GIV (control)	6	0.638±0.0012	0.0004	0.637	0.639		
Three	G1 (3%)	6	0.512±0.0010	0.0004	0.510	0.513	93123.06	0.001*
	GII (5%)	6	0.443±0.0006	0.0002	0.442	0.443		
	GIII (10%)	6	0.402±0.0009	0.0004	0.401	0.403		
	GIV (control)	6	0.658±0.0008	0.0003	0.657	0.659		
Four	G1 (3%)	6	0.474±0.0016	0.0006	0.473	0.476	118240.57	0.001*
	GII (5%)	6	0.401±0.0008	0.0003	0.400	0.402		
	GIII (10%)	6	0.355±0.0008	0.0003	0.354	0.356		
	GIV (control)	6	0.697±0.0008	0.0003	0.696	0.698		
Five	G1 (3%)	6	0.444±0.0013	0.0005	0.443	0.445	167791.08	0.001*
	GII (5%)	6	0.366±0.0008	0.0003	0.365	0.367		
	GIII (10%)	6	0.311±0.0012	0.0004	0.310	0.312		
	GIV (control)	6	0.721±0.0008	0.0003	0.720	0.722		

Pairwise comparisons highlighted significant differences between the control group and the others, with Group IV being less effective, emphasizing the enhanced microbicidal effectiveness of zirconia nanoparticle-modified groups (Table 4).

**Table 4:** Pairwise comparison of antimicrobial efficacy of Lactobacillus among all groups * Significant at the 0.05 level; the error term is mean square (error)=2.630E-7.

Pairwise Comparison	Mean Difference	Std. Error	P value	95% Confidence Interval	
				Lower Bound	Upper Bound
3% vs 5%	0.054	0.00029	0.001*	0.053	0.055
3% vs 10%	0.108	0.00029	0.001*	0.107	0.109
3% vs control	-0.175	0.00029	0.001*	-0.176	-0.175
5% vs 10%	0.0540	0.00029	0.001*	0.053	0.054
5% vs control	-0.230	0.00029	0.001*	-0.231	-0.229
10% vs control	-0.284	0.00029	0.001*	-0.285	-0.283

Evaluation of compressive strength

**Figure 8:**
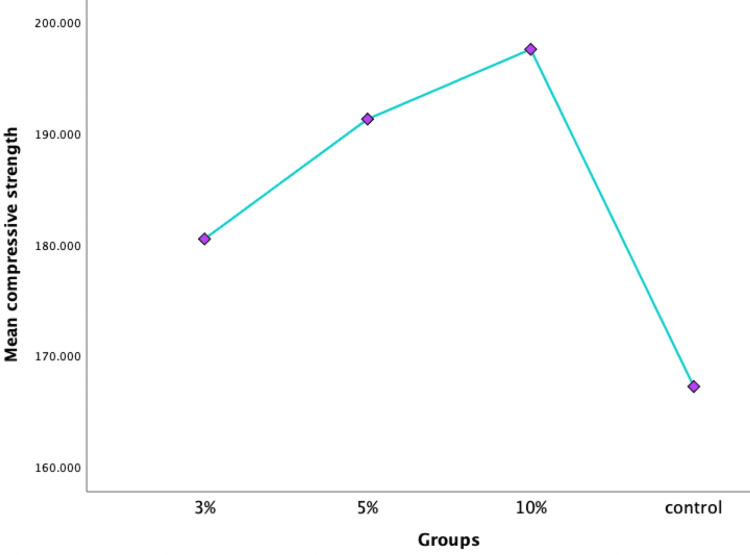
Compressive strength among all groups

Compression testing was conducted on the specimens, and the results were graphed linearly (Figure 8).

Significant differences between groups were found using one-way ANOVA (p < 0.05) (Table 5).

**Table 5:** Comparison between groups for the evaluation of compressive strength * Significant at 0.05; the p-value was derived by one-way analysis of variance (ANOVA).

Groups	N	Mean ± Std. Deviation	Std. Error	95% Confidence Interval for Mean		F value	P value
				Lower Bound	Upper Bound		
G1 (3%)	12	180.48±1.024	0.295	179.83	181.13	2473.69	0.001*
GII (5%)	12	191.25±0.524	0.151	190.91	191.58		
GIII (10%)	12	197.52±0.755	0.218	197.04	198.00		
GIV (control)	12	167.22±1.235	0.356	166.44	168.00		

Tukey's post hoc test highlighted performance disparities, with the modified groups surpassing the control group (p < 0.05). Furthermore, Group III showed notable superiority in compressive strength compared to the others (p < 0.05) (Table 6).

**Table 6:** Pairwise comparison for the evaluation of compressive strength * Significant at 0.05; the p-value was derived from Tukey's posthoc test.

Pairwise Comparison	Mean Difference	Std. Error	P value	95% Confidence Interval	
				Lower Bound	Upper Bound
3% vs 5%	-10.766	0.377	0.001*	-11.775	-9.758
3% vs 10%	-17.041	0.377	0.001*	-18.050	-16.033
3% vs control	13.258	0.377	0.001*	12.250	14.266
5% vs 10%	-6.275	0.377	0.001*	-7.283	-5.266
5% vs control	24.025	0.377	0.001*	23.016	25.033
10% vs control	30.300	0.377	0.001*	29.291	31.308

## Discussion

GICs have been utilized for over 50 years and are widely acknowledged as dental restorative materials with diverse applications [11]. Also, GIC is preferred over resin cement in various dental applications due to biocompatibility as it ensures minimal cytotoxicity and reduced risk of allergic reactions or tissue irritation, promoting patient comfort and long-term oral health. GICs are particularly favoured in patients with a high risk of decay due to their fluoride release and their ability to chemically and micromechanically bond to dental structures. GIC serves multiple purposes including luting cement, filler, and liner. However, their low mechanical strength and brittle nature are commonly cited as significant drawbacks. Fleming et al. suggested that air inclusion during the mixing process, leading to pores in the set cement, may contribute to the decrease in compressive strength of GIC [12]. Various attempts have been made to rectify these deficiencies in mechanical and physical properties through the integration of different filler particles [13]. Ideally, such filler inclusion should enhance the properties of GIC without compromising their inherent characteristics. Numerous approaches have been explored to enhance the properties of GIC while each strategy has shown some improvement in mechanical properties, these results have not yet been applied clinically. Moreover, even with fluoride present in GIC, the quantity released is inadequate to confer anti-cariogenic properties, thus leaving secondary caries as an ongoing concern [14]. Furthermore, attempts to add antibacterial agents have often compromised the fundamental physico-mechanical characteristics of the material.

Nanotechnology offers avenues for enhancing mechanical properties, augmenting antimicrobial features, and optimizing the biocompatibility and biomineralizing attributes of the materials. Recently, various nanoparticles such as titanium dioxide, nano-hydroxyapatite, silver particles, fluorapatite, N-vinylpyrrolidone, and zirconia have been incorporated to bolster the properties of GIC [15]. Nano-sized particles were chosen in this study, as prior research has indicated that larger particles could marginally diminish the physical properties of GIC. Besides particle size and distribution, how particles are integrated into the cement matrix also contributes to enhanced properties. Prentice et al. concluded that as the number of smaller particles increases, the strength and longevity of restorations improve as well [16]. While nanoparticles were utilized, this study opted to add particles with micrometre dimensions to the powder, as larger particle sizes can enhance resistance against pressure forces.

Zirconia material possesses mechanical resistance, toughness, and biocompatibility, along with aesthetic qualities resembling tooth colour. It acts as an outstanding supplement for boosting mechanical characteristics and can enhance aesthetics by diminishing the opacity of traditional GIC or modified substances [17,18]. Additionally, zirconium oxide particles play a vital role in mitigating porosity in cement. Their incorporation displaces air pockets during spatulation, leading to a denser structure and reduced porosity formation, thereby enhancing cement quality and performance. Nevertheless, there is a research gap concerning how adding varying concentrations of nanoparticles to conventional GIC affects their physical properties. Therefore, the current study was planned at different concentrations. In this research, zirconia nanoparticles were synthesized using a plant extract by excluding external chemicals such as reducing agents and stabilizers, thereby blending the extract with the zirconium salt solution. Consequently, the phytochemicals in the extract act as stabilizers for producing zirconia nanoparticles, which are recognized for their low toxicity. The antibacterial activity of zirconia nanoparticle-modified GIC was assessed against *S. mutans* and *Lactobacillus*, demonstrating pronounced antibacterial activity compared to conventional GIC. Tiwari et al.'s study revealed that zirconia-reinforced GIC exhibited maximum antibacterial activity against *S. mutans* compared to conventional GIC [19]. Similarly, another study by Surabhilakshan et al. indicated that zirconia-reinforced GIC with fluoride release is a promising material for restorations with anticarcinogenic properties [20]. Feiz et al. reported that zirconomer exhibited the highest fluoride release and maximum antibacterial activities [21]. Additionally, Kukreja et al. demonstrated that zirconomer exhibited higher fluoride release compared to GIC at all time intervals, consistent with our study [22].

In the present study, GIC infused with zirconia nanoparticles outperformed conventional GIC in terms of both antimicrobial properties and compressive strength. Gu et al. discovered that integrating hydroxyapatite and zirconia particles uniformly in the GIC matrix improved mechanical properties such as strength, modulus, and hardness due to the presence of zirconia nanoparticles [23]. Alobiedy et al. observed that incorporating zirconia nanoparticles improves the mechanical characteristics of GIC [24]. Similarly, Melo et al. demonstrated that adding 8.5 wt% zirconium oxide to GIC effectively enhances the quality of dental restorations [25]. Additionally, Venugopal et al. stated that modifications of GIC with zirconia nanoparticles substantially improved the physio-mechanical properties [26]. Fazelian et al. concluded that the addition of 15%w zirconium oxide particles in glass ionomer increases the mean compressive strength [27].

The graph depicting the compressive strength of modified GIC clearly illustrates a linear trend with the incorporation of zirconia nanoparticles, indicating the cement's resistance until fracture without any bending of the curve as the load increases. This underscores the enhanced durability and resistive nature of the modified cement. Additionally, SEM serves as a valuable tool for assessing surface morphology, filler size, uniformity, and porosity distribution [12]. The enhanced mechanical properties noted in this investigation are credited to the accurate incorporation of zirconia charges and the uniform distribution of glass particles, thereby boosting the material's resilience and capacity to endure occlusal forces. Consequently, GICs modified with zirconia nanoparticles exhibit favourable properties suitable for use in restorations subjected to high levels of stress areas. It is imperative to acknowledge a limitation of this in vitro study, as it does not entirely replicate oral conditions, thereby potentially affecting the prediction of clinical outcomes. The strength of the restorative materials within the oral cavity is affected by various factors, including the presence of a salivary film and the impact of specific dietary items, making it difficult to accurately replicate these conditions in laboratory experiments. Further studies may aim to conduct in vivo research to mimic oral conditions more realistically, exploring factors such as material discolouration, surface roughness, and the effectiveness of protective coatings over varied immersion durations. These endeavours seek to enhance the development of restorative materials by addressing the current limitations highlighted in this study.

## Conclusions

Despite the study's limitations, it's evident that the 10% green-mediated zirconia-modified GIC showed superior compressive strength and antimicrobial activity compared to conventional formulations. This enhancement could significantly improve the durability and efficacy of dental restorations, contributing to patient satisfaction. Future research is needed to address these limitations and enhance our understanding of GIC properties. Implementing zirconia nanoparticle-modified GIC could offer clinical benefits, including improved properties and potentially establishing them as leading dental materials.
